# Graft Polymers Derived from Pharmaceutically Active Choline-Based Ionic Liquid Monomers: Dual Incorporation of Ampicillin and Cloxacillin

**DOI:** 10.3390/ijms26199415

**Published:** 2025-09-26

**Authors:** Aleksy Mazur, Dorota Neugebauer

**Affiliations:** Department of Physical Chemistry and Technology of Polymers, Faculty of Chemistry, Silesian University of Technology, 44-100 Gliwice, Poland; aleksy.mazur@polsl.pl

**Keywords:** choline methacrylate, biofunctionalized monomeric ionic liquid, ionic graft copolymers, ionic conjugate, ampicillin, cloxacillin

## Abstract

This study reports the synthesis and characterization of well-defined ionic graft conjugates acting as drug delivery systems, based on monomeric ionic units derived from choline methacrylate (TMAMA) biofunctionalized with the anions of ampicillin (AMP) or cloxacillin (CLX). Using the “grafting from” technique with multifunctional macroinitiators, the density of side chains was precisely defined, and the length of side chains was well-controlled during polymerization. The resulting ionic conjugates featured the regulated content of ionic fractions with drug anions reaching up to 55% and drug content up to 48–70% for AMP, 27–65% for CLX, and 47–79% for (CLX + AMP). The drug release behavior was evaluated under physiological conditions using a dialysis method. The ionic conjugates demonstrated release efficiencies of 70–93% for CLX (5–16 µg/mL), 69–98% for AMP (12–13 µg/mL) in single systems, and 61–73% for CLX + AMP (10–15 µg/mL) in dual systems. Additionally, polymer surface properties were evaluated via water contact angle measurements (WCA = 30–54°). In an aqueous solution, the polymer self-assemblies appeared to be nanosized particles (90–360 nm). The results demonstrate that the synthesized TMAMA-based graft copolymers act as effective ionic conjugates and dual drug systems, offering a promising platform for controlled and multi-drug delivery applications.

## 1. Introduction

Traditional antibiotic delivery methods, including oral administration, intramuscular injections, and intravenous infusion, face several limitations, including low bioavailability, rapid metabolic degradation, and the need for frequent dosing. These issues often lead to adverse side effects and make it difficult for patients to adhere to treatment, ultimately reducing the therapeutic effectiveness [1,2]. At the same time, antibiotic resistance is an escalating global health concern, with once-treatable infections becoming increasingly difficult to manage. To address this, combination antibiotic therapy is often employed to combat multidrug-resistant bacterial strains, providing enhanced therapeutic outcomes and lowering the risk of treatment failure. Common examples include the combination of amoxicillin with clavulanic acid, ampicillin with cloxacillin, ampicillin/cloxacillin with metronidazole, isoniazid with rifampicin, piperacillin with tazobactam, and trimethoprim with sulfamethoxazole [3,4]. A promising strategy to overcome these challenges is the use of polymeric materials for the delivery of biologically active compounds. Such systems can protect drugs from premature degradation, improve solubility and bioavailability, and enable the controlled or sustained release. These features not only enhance the overall effectiveness of conventional therapies but also reduce dosing frequency, minimize side effects, and improve patient adherence [5,6,7]. Different approaches have been investigated; drugs can be encapsulated in amphiphilic copolymers [8,9,10,11,12,13], conjugated to hydrophilic polymers [14,15,16,17], or incorporated into ionic liquids (ILs) [18,19,20,21,22,23,24]. Owing to their physicochemical properties, ILs have found numerous biomedical applications [25,26], including the ability to interact with negatively charged drug anions [27,28,29,30,31,32,33,34]. This property enables the synthesis of polymerizable IL monomers, which can serve as building units for ionic drug–polymer conjugates [35,36]. Poly(ionic liquid)s (PILs) carrying pharmaceutical anions can be synthesized via two strategies: (1) direct polymerization of the IL-based monomers with drug anions forming PILs, and (2) post-polymerization ion exchange to incorporate drug anions into the polymer. Guanidinium-based polycations have been developed to carry ionic forms of ampicillin and nicotinic acid [37]. One of the most promising IL monomers is [2-(methacryloyloxy)ethyl]trimethylammonium chloride (TMAMA/Cl), which is chemically stable, water-soluble, and capable of undergoing ion exchange to introduce functional counterions [38,39]. Choline-based polymers are known for their biocompatibility and enhanced drug bioavailability [40,41]. Linear and grafted PILs derived from TMAMA have already been reported for the delivery of various pharmaceutical anions, including mefenamate [42,43], sulfacetamide, salicylate [40,44], *p*-aminosalicylate, clavulanate [39,45], piperacillin, and fusidate [39,46]. Recent strategies have focused on functionalizing TMAMA monomers with pharmaceutically active anions before polymerization. These monomers have been used to synthesize both linear [38,47,48] and grafted PILs [44,49,50], designed for the controlled delivery of various pharmaceutical anions, including fusidate, cloxacillin (CLX), *p*-aminosalicylate, and ampicillin (AMP). However, the extended research toward TMAMA-based carriers containing β-lactam antibiotics was limited to copolymers with linear architectures [48,51]. Additionally, only a limited number of studies have investigated the use of polymer-based carriers in which AMP or CLX are either loaded into polymeric systems or covalently conjugated to polymers [52,53,54,55]. These antibiotics, AMP as a broad-spectrum amino-penicillin antibiotic effective against a wide range of Gram-positive and Gram-negative bacteria, and CLX, also a β-lactam antibiotic, which is resistant to β-lactamase enzymes and particularly active against penicillinase-producing streptococci [56,57,58], are commonly used in clinical practice to treat infections of the respiratory tract, ear, nose, and throat, urinary tract, skin, soft tissue, and gastrointestinal system. Notably, they are generally well-tolerated, with rare occurrences of adverse reactions, mostly allergic in nature. Their combination exhibits synergistic antibacterial activity, which is represented by commercially available formulations, such as AmpicloxCare, Ampiclox, and Cloxam, but these products do not utilize polymer-based delivery systems.

In this study, we report new graft copolymer ionic conjugates incorporating the β-lactam antibiotics AMP and CLX as pharmaceutical counterions. These copolymers were synthesized via atom transfer radical polymerization (ATRP) using a “grafting-from” approach, starting from macroinitiators and TMAMA comonomers functionalized via ion exchange with AMP or CLX. Depending on the monomer composition, the systems were tailored to carry either single (AMP or CLX) or dual drugs (AMP/CLX). These systems were designed to offer controlled drug content and were subjected to in vitro release through anion exchange in phosphate-buffered saline (PBS, 37 °C) as the physiological environment. Previously, the linear PIL conjugates with AMP and CLX [48] have been investigated, whereas the present work aims to expand the combined AMP/CLX dual-drug systems based on ionic polymer–drug conjugates by focusing on grafted architectures, which have been applied earlier for another dual-drug system containing CLX and fusidate, showing good biocompatibility with human bronchial epithelial cells [49]. The current research aims to explain the influence of the polymer structure, (i) the ionic unit content tuned by the initial TMAMA-to-comonomer ratio; (ii) the side-chain length adjusted via the monomer-to-macroinitiator ratio; and (iii) the grafting density defined by the number of initiating sites on the macroinitiator, on the resulting polymers’ physicochemical properties, drug loading, and release behavior as the crucial correlations for widespread ionic graft polymers in drug delivery systems.

## 2. Results and Discussion

**Figure 1:**
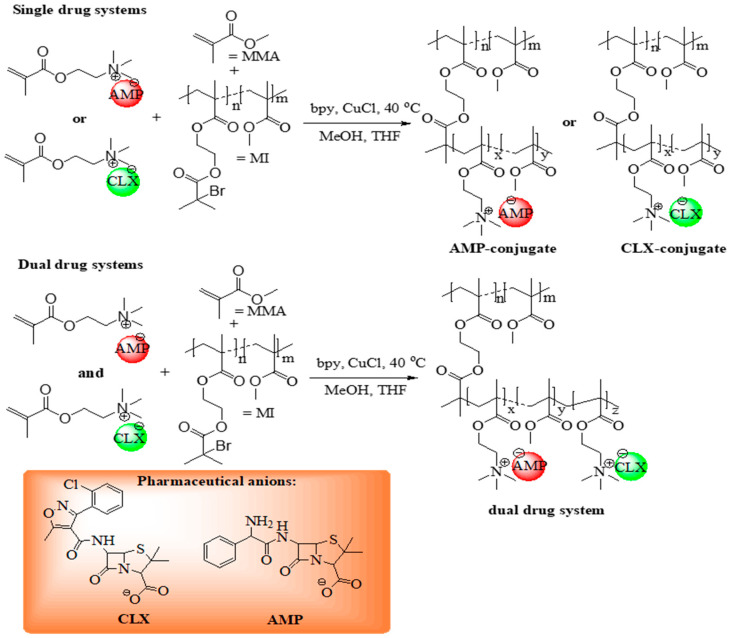
Schematic route for synthesis of ionic graft conjugates, presented as single and dual systems carrying AMP or/and CLX anions, derived from biofunctionalized choline methacrylate.

The monomeric ILs, [2-(methacryloyloxy)ethyl]trimethylammonium cloxacillin (TMAMA/CLX) and [2-(methacryloyloxy)ethyl]trimethylammonium ampicillin (TMAMA/AMP) [48,49] were utilized in ATRP via the “grafting from” strategy to synthesize well-defined graft copolymers (Figure 1) as drug delivery systems (DDS). These former single-ionic drug conjugates were obtained by copolymerizing the TMAMA/CLX (series C1–C4) or TMAMA/AMP (series A1–A4) with MMA at two feed ratios (25:75 and 50:50) in the presence of MIs, using a CuCl/bpy catalytic system at 40 °C in a MeOH/THF mixture (Table 1). The terpolymerization of both ionic monomers TMAMA/CLX and TMAMA/AMP with MMA in the ratios equal to 12.5:12.5:75 and 25:25:75 was applied to achieve dual-ionic drug conjugates (series AC1–AC4, Table 2).

**Figure 2:**
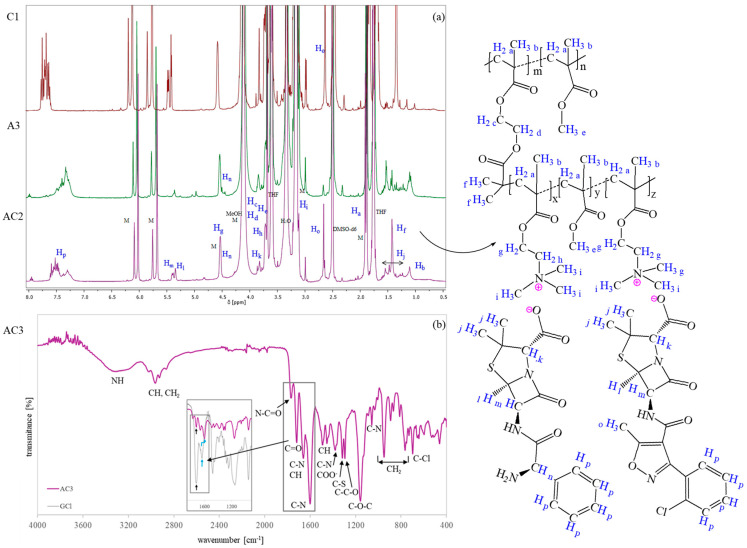
^1^H NMR spectra of reaction mixtures containing ionic conjugates bearing CLX or AMP and both CLX and AMP anions (**a**), where signals corresponding to unreacted TMAMA and MMA monomers are labeled as “M”. FT-IR spectrum of dual-drug system (**b**).

In both cases, the successful formation of PIL side chains was confirmed by ^1^H NMR spectroscopy (Figure 2a), which revealed characteristic signals of AMP and CLX (J-P) [44,48,59,60] and TMAMA moieties (intensive H and I signals corresponding to –CH_2_–N^+^– and –N^+^(CH_3_)_3_ protons). In the spectrum of the dual-drug systems, distinct signals corresponding to the methyl group on the isoxazole ring of CLX (O) and assigned to the –CH–NH_2_ group of AMP (N) were observed. The latter overlapped with the G signal originating from –CH_2_–O. Additionally, signals A (–CH_2_–), B (–CH_3_), and E (–O–CH_3_) exhibited an increased intensity relative to the MI, supporting the successful grafting process. Monomer conversion was determined by the ^1^H NMR analysis of the post-reaction mixture, based on the integration of residual TMAMA (6.1 ppm) and MMA (6.0 ppm) peaks, referenced against the fixed pyrene signal (8.19–8.22 ppm) serving as an internal standard. Then, a degree of polymerization was estimated as the product of the monomer conversion and the initial molar ratio between the monomer and macroinitiator. All ^1^H NMR spectra are shown in Figures S4–S6. Figure 2b presents the FT-IR spectrum of the dual-ionic conjugate, showing characteristic peaks attributed to polymethacrylates [61], including the stretching vibration of C=O at 1767 cm^−1^, CH bending at 1450 cm^−1^, asymmetric stretching of the C–O–C bond at 1160 cm^−1^, stretching vibrations of the ester group (–COO^−^) at 1300 cm^−1^, and rocking mode vibrations of the –CH_3_ group at 766, 890, and 950 cm^−1^. Additionally, an intense absorption band at 1600 cm^−1^ corresponding to the trimethylammonium group is observed. Several bands can also be attributed to the incorporated drug anions [38,62,63], including N–C=O and C–S stretching in the β-lactam ring (1767, 1320 cm^−1^), C–N stretching (1660 cm^−1^), aromatic C–H bending (1660, 1490 cm^−1^), COO¯ asymmetric stretching (1380 cm^−1^), C–O (1320 cm^−1^), C–O–C (1160 cm^−1^), C–N (1060 cm^−1^), and C–Cl (750 cm^−1^) vibrations. The spectrum was also compared to that of analogous copolymers containing chloride anions (GCl [49]) instead of drug anions. Typically, the carbonyl group shows a higher absorbance than the C–N group; however, in the dual-drug conjugates, the C–N band exhibited both enhanced intensity and a shift in the wavenumber. This suggests the formation of ionic bonds between drug molecules and polymers. The FT-IR data for dual-drug systems with ampicillin and cloxacillin are well-correlated with the FT-IR spectra of single systems with ampicillin or cloxacillin (Figure S1), which contained comparative reference bands.

The polymeric backbones, P(MMA-*co*-BIEM), were characterized by two various degrees of polymerization (DP_n_ = 229 vs. 214) and the numbers of initiating units (63 vs. 103). The grafting degree (DG) was controlled by the molar content of initiating groups (28 mol% vs. 48 mol%), corresponding to 63 and 103 grafting sites, respectively, which directly determined the number of side chains (n_sc_). The grafted side chains, P(TMAMA/CLX-*co*-MMA) and P(TMAMA/AMP-*co*-MMA), differed in several parameters, including the molar fraction of TMAMA (single systems: F_TMAMA/CLX_ = 11–30 mol%, F_TMAMA/AMP_ = 22–52 mol%; dual systems: F_TMAMA/CLX_ = 7–19 mol%, F_TMAMA/AMP_ = 13–36 mol%) and the degree of polymerization of the side chains (single systems: DP_sc_ = 21–60, dual systems: DP_sc_ = 27–44). For clarity, the polymer samples are labeled with the letters A, C, or AC, indicating the presence of AMP, CLX, or both drugs, respectively.

Due to the solubility of IL monomers in MeOH and MIs in THF, a MeOH/THF mixture was used as the reaction medium. A higher MeOH content ensured the complete dissolution of the IL monomer and excluded cross-linking, while the THF amount was carefully adjusted to prevent precipitation. The proper solvent ratio also helped suppress the transesterification of the ionic units to MMA. However, the increased solvent volume slowed polymerization, requiring extended reaction times of up to 20 h to achieve sufficient monomer conversion. In general, reactions involving TMAMA/AMP required a higher solvent volume to prevent gelation compared to TMAMA/CLX systems, which became deactivated at elevated solvent ratios. For the synthesis of dual-drug systems, solvent amounts were optimized accordingly. Specifically, for reactions AC3–AC4 with a higher initial content of ionic monomer relative to MMA, the MeOH:TMAMA ratio was increased slightly at the maintained ratio of MeOH:THF. This adjustment ensured good solubility of the reaction mixture and helped prevent side reactions.

**Figure 3:**
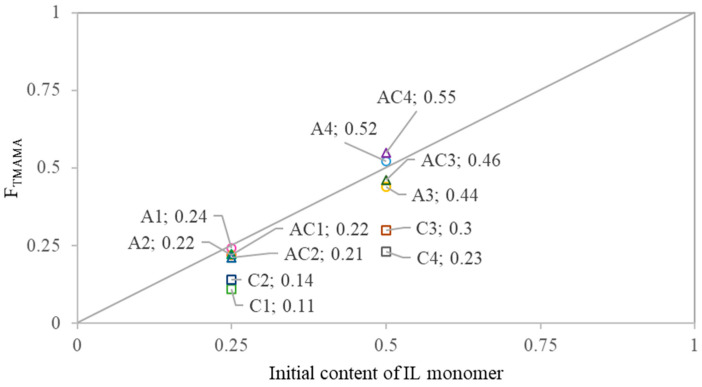
Copolymer composition vs. initial ionic monomer feed composition.

The copolymer composition was compared to the initial monomer feed to assess the reactivity of IL monomers toward MMA (Figure 3). All AMP conjugates showed a similar reactivity of TMAMA/AMP and MMA, resulting in side chains with compositions close to the initial feed. In contrast, all CLX conjugates exhibited a notable depletion of F_TMAMA_ in the copolymer relative to the feed, indicating lower reactivity of the ionic monomer than MMA. Dual-drug conjugates generally showed a similar incorporation of IL monomers, as observed in the AMP conjugates. The comparison of TMAMA/CLX and TMAMA/AMP reactivities revealed that CLX-based monomers were incorporated into the PIL side chains less efficiently than their AMP-based counterparts. TMAMA units conjugated with CLX represented approximately 30–38% of the total F_TMAMA_, suggesting a lower reactivity and incorporation efficiency of TMAMA/CLX compared to TMAMA/AMP.

The presence of ionic drugs and PIL side chains could cause a steric hindrance and destabilize the control of the reaction due to the charge effects on propagating radicals or the catalytic complex. Despite these potential challenges, the dispersity indices (Ð) remained low for the series of CLX conjugates (1.19–1.28), suggesting that the incorporation of ionic units bearing CLX anions did not disturb the control over ATRP, yielding monodispersed polymers. Higher values were observed for the series of AMP conjugates (1.52–1.63) because of the higher reactivity of TMAMA/AMP than TMAMA/CLX, which provided its faster propagation. Additionally, the broadened SEC peaks can be the result of potential aggregation in water, where anion dissociation is less efficient than in DMF. In case of the dual-drug systems (series AC), the incorporation of both types of TMAMA monomers resulted in the polymers with the highest dispersity (1.43–1.76) as the combined effect of both TMAMA units with different pharmaceutical anions incorporated with various rates into the side chains. Discrepancies between the number-average molecular weights determined from monomer conversion (M_n,NMR_) and those obtained via SEC (M_n,SEC_) are attributed to the SEC calibration, which was based on linear PEO standards with different hydrodynamic volumes compared to the studied non-linear polymers. In the series of AMP conjugates, M_n,NMR_ values were higher than M_n,SEC_, which are apparent values for graft copolymers, and these differences were reduced with the increase in content of ionic fractions, possibly due to increasing electrostatic repulsions and the formation of rod-like structures. In contrast, for CLX-conjugates, M_n,NMR_ values were lower than M_n,SEC_, and differences of M_n,NMR_ vs. M_n,SEC_ were increased with the TMAMA content, potentially because dissolution in DMF leads to different behaviors of PIL side chains and provides more expanded structures. Therefore, these ionic conjugates may have eluted faster than PEO standards, resulting in an overestimation of their molecular weights. In the dual systems, particularly for AC4 with a higher grafting density and the highest ionic content and drug content, the M_n,NMR_ > M_n,SEC_ relationship was observed similarly to series A, whereas for the other AC conjugates, the molecular weights appeared to be overestimated by SEC for series C. SEC chromatograms of the polymers are shown in Figure S3, but the direct comparison of MI vs. polymer conjugates was difficult due to differences in their solubility, requiring analyses in different solvents. Furthermore, the grafted polymer topology led to the determination of apparent molecular weight due to the use of linear polymers as calibration standards.

**Figure 4:**
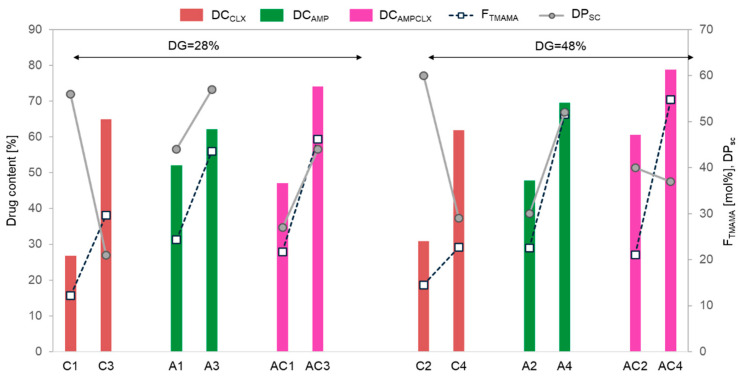
Correlation of ionic fraction and drug content with side-chain length and grafting density in copolymers. The colored bars corresponding to drug contents (DC_CLX_, DC_AMP_, and DC_AMPCLX_) are related to the left y-axis, whereas the connected points representing ionic fraction content (F_TMAMA_ by squares) and side-chain length (DP_sc_ by circles) are related to the right y-axis.

The drug content (DC) represented by the percentage of ionic drugs incorporated into the copolymer and assessed by UV-Vis spectroscopy ranged between 48–70% and 27–65% for single AMP- and CLX-based systems, respectively. In the dual systems, the total drug content (AMP+CLX) varied from 47% to 79%. Generally, for all single- and dual-drug conjugates, the DC significantly increased with ionic content regardless of the DG (Figure 4). AMP conjugates, characterized by longer side chains, exhibited a higher DC than copolymers with shorter grafts, whereas CLX conjugates showed an inverse correlation, likely due to the higher steric hindrance of CLX moieties. In the case of dual-drug systems, the DC tended to be higher in copolymers with a greater F_TMAMA_ and longer, loosely distributed side chains (AC1 vs. AC3). However, in densely grafted brushes, such as AC2 and AC4, the DC was influenced more by the F_TMAMA_ content than by DP_sc_. An exception was noted for A1 and A3, which exhibited similar AMP content regardless of F_TMAMA_ or DP_sc_.

**Figure 5:**
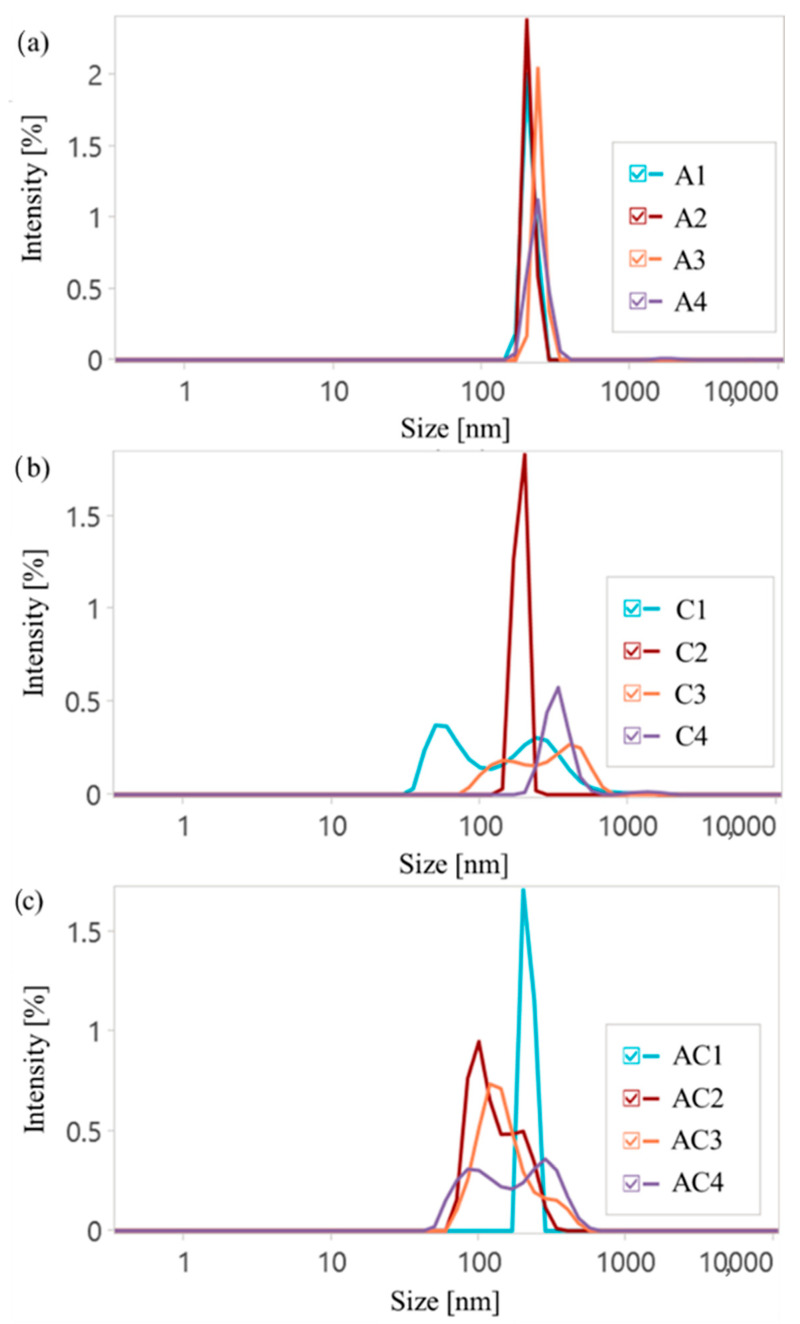
DLS histograms of AMP conjugates (**a**), CLX conjugates (**b**), and dual-drug systems (**c**).

Dynamic light scattering (DLS) measurements were performed to determine the hydrodynamic diameters (D_H_) and polydispersity indices (PDI) of drug delivery polymer particles, as well as to show the influence of ionic nature on the self-assemblies formed in an aqueous solution (Figure 5, Table S1). In general, the ionic content in the copolymers was a key factor contributing to increased D_H_, as a higher charge density promoted the expanding of the polymeric chains in aqueous media. Moreover, a higher DC is beneficial for additional interactions and could provide the formation of bigger particles. However, there was no correlation between the DG and D_H_, with the exception of the CLX series, where a stronger influence was observed for copolymers with a lower content of ionic fractions (F_TMAMA_ < 15%). This is consistent with findings showing that most drug delivery systems exhibited sizes in the range of 180–360 nm (single systems) and 90–300 nm (dual systems).

All AMP conjugates demonstrated similar and narrow PDI values, indicating uniform self-assembly and the increase in particle sizes with F_TMAMA_, likely due to the stronger electrostatic repulsion and higher expanding of the polymeric chains. For CLX conjugates, a similar trend was observed, but these particles were notably larger than those containing AMP, likely due to a lower DC, which reduced interactions with the polymer and resulted in looser packing. In the case of densely grafted brushes (C2 and C4), lower PDI values were observed. This was likely due to stronger ionic interactions, which promoted a more uniform self-assembly compared to loosely grafted systems C1 and C3. Furthermore, for copolymers with both a high DG and high DC (A4, C2, and C4), small fractions of aggregates appeared, likely resulting from the combined effects of the high charge density and steric hindrance. Dual-drug systems usually exhibited two separate size populations. The overall complexity of dual-drug conjugates was reflected in their higher PDI values. An exception was noted for AC1, in which the combination of a low DC and low DG resulted in reduced electrostatic and steric hindrance, yielding uniform particle sizes and a significantly narrower PDI. Moreover, results indicate that the investigated C1, AC2, AC3, and AC4 copolymers form both micellar-like assemblies and their aggregates. The observed bimodal size distributions reflected the coexistence of two particle populations: smaller, more compact assemblies as individual micelles and larger, secondary aggregates formed by micelle–micelle associations or interchain interactions. The ionic polymers can promote such aggregation behavior via electrostatic interactions and counterion-mediated bridging, which may favor the formation of a small fraction of larger aggregates.

**Figure 6:**
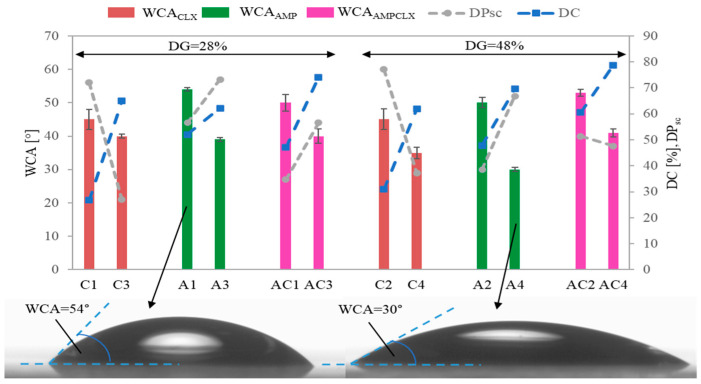
Correlation of WCA with DC, DP_sc_, and DG of copolymers, with the representative sessile drop images for A1 (lowest wettability) and A4 (highest wettability). The colored bars corresponding to water contact angles (WCA_CLX_, WCA_AMP_, and WCA_AMPCLX_) are related to the left y-axis, whereas the connected points representing drug content (DC by squares) and side-chain length (DP_sc_ by circles) are related to the right y-axis.

Wettability measurements were performed using goniometry via the sessile drop method, with water droplets deposited on polymer films spin-coated onto glass substrates (Table S2). The water contact angle (WCA) was used as an indicator of the hydrophilicity of the ionic conjugates. Across all copolymers, an increase in the DC correlated with the enhanced wettability of the polymer films, independently of DG or DP_sc_ (Figure 6). However, it can be observed that copolymers with a high DG (48%) were more sensitive to changes in the WCA. Notably, samples A4 and C4, with densely grafted side chains and high ionic contents, exhibited the greatest wettability. Interestingly, dual-drug systems showed a similar or slightly reduced hydrophilicity compared to single-drug systems (WCA = 40–53° vs. 30–54°).

**Figure 7:**
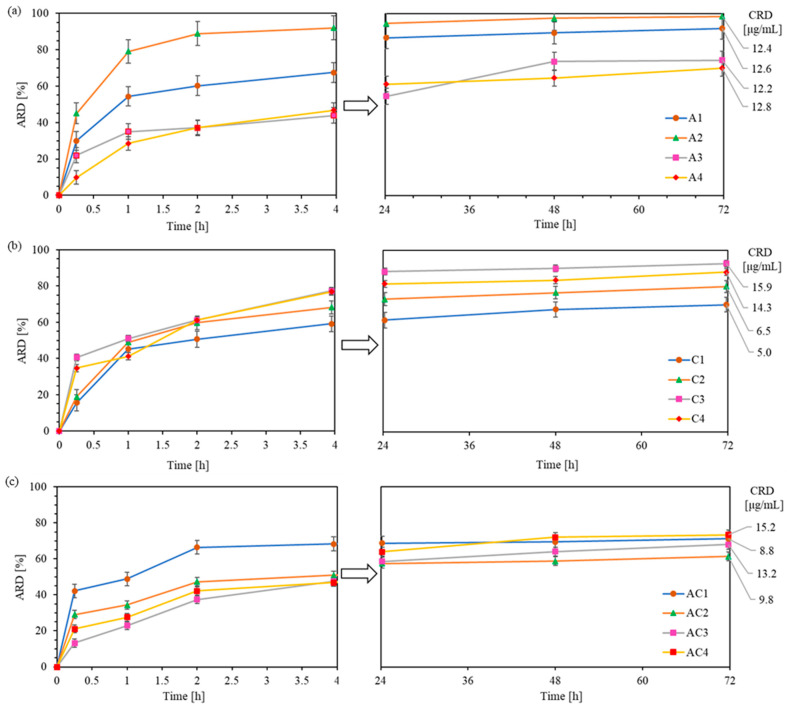
Release profiles for AMP in single-drug systems (**a**), CLX in single-drug systems (**b**), and CLX/AMP in dual-drug systems (**c**), with indicated final concentrations of released drugs.

In vitro drug release studies were conducted to monitor the exchange of CLX or/and AMP anions in polymer with phosphate anions from PBS, using the dialysis method under physiological conditions (pH 7.4, 37 °C) over 72 h. Drug release from the polymer samples was analyzed by UV–Vis spectroscopy at specific time intervals and expressed as the percentage of released drug (ARD) and the concentration of released drug (CRD) (Table S3). One of the goals of DDS is to prolong the effective drug release in time. In general, all conjugates presented here demonstrated sustained drug release for up to 3 days. Exceptions were for A2 and AC1 which showed exceptionally fast and effective drug delivery within 4 h, followed by a near-plateau up to 72h (additional 3-6% of drug release). In the case of CLX conjugates, burst release occurred within the first 4 h, leading to high ARD values (59–78%), followed by a slow and sustained additional release of 10–15% up to 72 h (Figure 7b). Regarding AMP conjugates, the release was less intense, reaching ARD values of 44–67% in 4 h and releasing an additional 23–30% by 72 h (Figure 7a). The cumulative ARD from dual-drug carriers were similar to those of series A, ranging between 46 and 68% at 4 h with an additional 11–26% of drug released up to 72 h (Figure 7c).

It can be observed that, in systems containing AMP and high ionic contents (A3–A4), drug release was notably slower within the first hour compared to CLX conjugates (28–35% vs. 41–51%). This suggests that the AMP exchange efficiency can be more effectively controlled than that of the CLX by adjusting the ionic content. A similar trend was observed in dual-drug systems, where AC3 and AC4 showed a slower initial release compared to AC1 and AC2 (23–28% vs. 34–48%). These findings suggest that CLX exhibits weaker interactions with the polymer than AMP in the systems characterized by high ionic contents, likely due to its greater steric hindrance and lower DC, resulting in a faster initial release.

Drug release profiles were analyzed using several kinetic models. The samples did not follow zero-order kinetics, demonstrating a concentration-dependent and diffusion-driven drug release by a plots well-fitted to first-order and Higuchi models, with the linear correlation coefficients of R^2^ = 0.62–0.92 and 0.6–0.94, respectively (Table S4, Figure S2). The nature of the diffusion process of released drugs via ion exchange was characterized by the Korsmeyer–Peppas model with high fitting coefficients (R^2^ = 0.90 − 0.98), indicating a quasi-Fickian diffusion described by the release exponents *n* < 0.45. An exception was observed for samples C1 and C2, where *n* ≈ 0.45, suggesting the ideal Fickian diffusion from cylindrical micelles, whereas sample A4 exhibited the *n* > 0.5 typical for non-Fickian processes as non-ideal by Fick’s law, where the release was probably governed by a combination of the diffusion and swelling of the micellar structure.

The final results after 72 h showed that loosely grafted copolymers with conjugated CLX demonstrated the release of higher drug quantities from copolymers with shorter side chains and a higher ionic content (C1 vs. C3: 70% vs. 93%). However, densely grafted brushes C2 and C4 provided similar ARD values (80–85%) despite differences in DP_sc_ and F_TMAMA_ content. The CRD from both C3 and C4 was approximately 15 µg/mg, about twice as high as that from C1 and C2. In the case of AMP conjugates (ARD = 70–98%), a reverse trend was observed, with decreasing ARD values across F_TMAMA_ content despite a similar DG. This occurred in copolymers with longer side chains (A3 and A4), which tend to form more densely packed structures that more efficiently entrap large AMP molecules, thereby limiting their mobility. As a result, all AMP conjugates provided similar CRD values (ca. 12–13 µg/mg). For all dual-drug systems, ARD values were comparable (61–73%) regardless of the structural parameters of polymers, suggesting that the release efficiency was limited by AMP/CLX interactions. The presence of both drugs likely led to stronger drug–polymer binding or more stable self-assemblies hindering release. However, due to a higher DC, AC3 and AC4 exhibited a higher cumulative CRD than AC1 and AC2 (13–15 vs. 8–9 µg/mg).

The release profiles of the graft copolymers were compared with previously reported linear analogs. For CLX conjugates, the grafted systems showed superior release (ARD = 70–93%) compared to linear copolymers (ARD = 58–76%, [48]), allowing for higher CRD values from C3 and C4 (14–16 µg/mg vs. 10–13 µg/mg). In the case of AMP conjugates, the linear systems provided higher CRD values (15–25 µg/mg, [51]) due to a higher DC compared to grafted systems (12 µg/mg). For dual-drug systems, a lower ARD was observed in grafted carriers (60–70%) compared to linear analogs (90–100%), resulting in lower CRD values for AC3 and AC4 (9–15 vs. 13–23 µg/mg). In addition, in all linear systems, drug release was generally completed within 4 h, whereas most grafted systems exhibited a sustained and effective release lasting up to 72 h.

## 3. Materials and Methods

Methyl methacrylate (MMA) and 2-hydroxyethyl methacrylate (HEMA) from VWR (Gdansk, Poland) were dried using molecular sieves (type 4A, bulk density 640–670 kg/m^3^, Chempur, Piekary Śląskie, Poland). The compound [2-(methacryloyloxy)ethyl]trimethylammonium chloride (TMAMA/Cl), available as an 80% aqueous solution from Sigma-Aldrich (Poznan, Poland), was concentrated under a vacuum to produce a solid substance. Copper(I) chloride and copper(I) bromide (CuCl and CuBr, both with purity of 98%), from Fluka (Steinheim, Germany), underwent purification by being stirred in glacial acetic acid, then filtered and washed with both ethanol and diethyl ether, before drying under vacuum. Methanol (MeOH, PureLand, Stargard, Poland), diethyl ether (Chempur, Piekary Śląskie, Poland), *N*,*N*-dimethylforamide (DMF, POCH, Gliwice, Poland), deuterated dimethyl sulfoxide (DMSO–d6, VWR, Gdansk, Poland), 2,2′-bipyridine (bpy, VWR, Gdansk, Poland), ethyl 2-bromoisobutyrate (EBiB), α-bromoisobutyryl bromide, and (BIBB) 4,4′-dinonyl-2,2′-dipyridyl (dNbpy) were all purchased from Sigma-Aldrich (Poznan, Poland); tetrahydrofuran (THF, Eurochem BGD, Tarnow, Poland) and phosphate-buffered saline (PBS, pH = 7.4, Thermo Fisher Scientific, Warsaw, Poland) were used directly without further processing. The pharmaceutical reagents used without prior purification included ampicillin sodium salt (NaAMP, Sigma Aldrich, Poznan, Poland) and cloxacillin sodium monohydrate (NaCLX, Alfa Aesar, Warsaw, Poland).

### 3.1. Functionalization of Choline-Based Ionic Liquid (TMAMA)

The biofunctionalization of TMAMA/Cl into TMAMA/CLX and TMAMA/AMP was carried out as previously described [48,49]. The ion exchange process with NaCLX was completed within 4 h at 20 °C, reaching an efficiency of 84%. Molar yield: 69%. In the case of NaAMP, the reaction carried out at 20 °C was terminated after 20 h, yielding an efficiency of 89% before a competing transesterification reaction could occur. Molar yield: 76%. ^1^H NMR data (DMSO-d_6_, 400 MHz, δ, and ppm): 1.80–1.95 (3H, –CH_3_), 3.07–3.15 (9H, –N^+^(CH_3_)_3_), 3.55–3.74 (2H, –CH_2_–N^+^–), 4.45–4.58 (2H, –CH_2_–O–), and 5.63–5.70 (2H, =CH_2_). CLX signals: 7.40–7.70 (4H, aromatic protons), 5.31–5.40 (1H, >CH–NH–), 5.28–5.35 (1H, N–CH–S–), 3.65–3.74 (1H,–CH–N–), 2.56–2.69, and 1.36–1.39 (9H, –CH_3_). AMP signals: 7.16–7.55 (5H, aromatic –CH), 5.25–5.39 (2H, >CH–N– and N–CH–S–), 4.31–4.49 (1H, –CH–NH_2_), 3.9–4.0 (1H, –CH–N–), and 1.27 and 1.41 (6H, –CH_3_).

### 3.2. Synthesis of Multifunctional Macroinitiators

Copolymers of methyl methacrylate and 2-(2-bromoisobutyryloxy)ethyl methacrylate (P(MMA-*co*-BIEM)) were employed as multifunctional macroinitiators (MIs). Their synthesis, based on copolymerization of MMA and HEMA and initiated by EBiB using catalytic complex CuBr/dNbpy in anisole (10 vol. % in relation to monomer) at 70 °C, and then esterification with BIBB (3-fold molar excess relative to hydroxyl groups in the copolymer) carried out at room temperature, were previously reported [45]. Details are given in Procedure S1. Two MIs with different contents of bromoester initiating groups, that is 28% (MI-1, M_n_ = 43,500 g/mol, and M_w_/M_n_ = 1.29) and 48% (MI-2, M_n_ = 36,400 g/mol, and M_w_/M_n_ = 1.26), were obtained. ^1^H NMR (DMSO-d_6_, 400 MHz, δ, and ppm) of P(MMA-*co*-BIEM): 3.77–3.48 (4H, –CH_2_–O–), 3.46–3.18 (3H, –OCH_3_), 1.94–1.86 (6H, –(CH_3_)_2_Br), 2.16–1.73 (2H, –CH_2_–, and backbone), and 1.1–0.5 (3H, –CH_3_,).

### 3.3. Synthesis of Ionic Graft Copolymers Containing Beta-Lactam Antibiotics

Graft copolymer containing CLX anions, P(MMA-co-(BIEM-*g*-P(TMAMA/CLX-*co*-MMA))) (example for C1): comonomers TMAMA/CLX (0.18 g, 0.33 mmol) and MMA (0.11 mL, 0.99 mmol), along with MeOH (0.45 mL), THF (0.34 mL), bpy (2.1 mg, 0.013 mmol), and MI-1 (3.6 mg, including 0.0066 mmol of initiating sites) were added to a Schlenk flask. The mixture was degassed through three freeze–pump–thaw cycles. An initial sample was taken before adding the CuCl catalyst (0.65 mg, 0.0066 mmol) to the mixture. The reaction was allowed to proceed for 20 h at 40 °C, after which it was terminated by exposure to air. The resultant polymer solution in THF was passed through a neutral alumina column to remove copper catalyst, precipitated twice using a 1:2 *v*/*v* THF-diethyl ether mixture, and subsequently dried under a vacuum. The CLX-based graft copolymers with various initial molar ratios of TMAMA/CLX to MMA (C1, C2: 25/75 and C3, C4: 50/50) and using both MIs (MI-1 for C1, C3 to obtain loosely grafted copolymers and MI-2 for C2, C4 to obtain densely grafted copolymers) were synthesized according to the same procedure.

Graft copolymer containing AMP anions, P(MMA-*co*-(BIEM-*g*-P(TMAMA/AMP-*co*-MMA))), (example for A2): comonomers TMAMA/AMP (0.16 g, 0.31 mmol) and MMA, (0.10 mL, 0.92 mmol), along with MeOH (0.48 mL), THF (0.36 mL), bpy (1.92 mg, 0.012 mmol), and MI-2 (2.4 mg, including 0.0062 mmol of initiating sites) were added to a Schlenk flask. The mixture was degassed through three freeze–pump–thaw cycles. An initial sample was taken before adding the CuCl catalyst (0.61 mg, 0.0062 mmol) to the mixture. The reaction was carried out for 20 h at 40 °C and then stopped by exposing it to air. Further steps of the procedure were the same as the above described for the copolymer with CLX anions. The AMP-based graft copolymers with various initial molar ratios of TMAMA/AMP to MMA (A1, A2: 25/75 and A3, and A4: 50/50) and using both MIs (MI-1 for A1, A3 to obtain loosely grafted copolymers and MI-2 for A2, A4 to obtain densely grafted copolymers) were synthesized according to the same procedure.

Dual-drug polymer conjugates containing both CLX and AMP anions, P(MMA-*co*-(BIEM-*g*-P(TMAMA/CLX-*co*-MMA-*co*-TMAMA/AMP))) (example for AC3): the comonomers TMAMA/CLX (0.2 g, 0.38 mmol), TMAMA/AMP (0.21 g, 0.38 mmol), and MMA (0.08 mL, 0.77 mmol), as well as MeOH (0.8 mL), THF (0.6 mL), bpy (2.4 mg, 0.015 mmol), and MI-1 (4.17 mg, including 0.0077 mmol of initiating sites) were placed into a Schlenk flask and degassed by three freeze–pump–thaw cycles. The initial sample was taken and the CuCl catalyst (0.8 mg, 0.0077 mmol) was introduced to the mixture. The reaction was carried out for 20 h at 40 °C and then stopped by exposing it to air. Further steps were carried out, like for the synthesis and purification of abovementioned examples. The AMP/CLX-based graft copolymers with various initial molar ratios of TMAMA/AMP to TMAMA/CLX to MMA (AC1, AC2: 12.5/12.5/75 and AC3, and AC4: 25/25/50) and using both MIs (MI-1 for AC1, AC3 to obtain loosely grafted copolymers and MI-2 for AC2, AC4 to obtain densely grafted copolymers) were synthesized according to the same procedure.

^1^H NMR data (Figure 2a) for side chains of dual-drug-containing polymers (DMSO–d6, 400 Hz, ppm): 7.16–7.70 (9H, –CH in aromatic rings of AMP and CLX), 5.20–5.50 (2H, >CH–NH– and N–CH–S– in β–lactam ring of CLX¯ and AMP¯), 4.50–4.58 (2H –CH_2_–O–, 1H –CH–NH_2_ in AMP¯), 4.00–4.30 (4H, –CH_2_–O–), 3.77–3.90 (2H, ¯OOC–CH–N– in thiazolidine ring of CLX¯ and AMP¯), 3.64–3.76 (2H, –CH_2_–N^+^–), 3.55–3.65 (3H, –O–CH_3_), 3.05–3.25 (9H, –N^+^(CH_3_)_3_), 2.59–2.74 (3H, –CH_3_ at isoxazole ring in CLX¯), 1.83–1.98 (2H, –CH_2_–), 1.40–1.60 (12H, –C(CH_3_)_2_ in thiazolidine ring of CLX¯ and AMP¯), and 0.6–1.2 (3H, –CH_3_).

FT–IR data (Figure 2b): NH (3300 cm^−1^), CH (2990–2950 cm^−1^), CH_2_ (1928 cm^−1^), N–C=O (1767 cm^−1^), C=O (1720 cm^−1^), C–N and =CH– (in lactam groups and aromatic rings at 1660 cm^−1^), C–N^+^ (1600 cm^−1^), =CH (aromatic rings at 1490 cm^−1^), CH (1450–1490 cm^−1^), C–N and COO¯ (1380 cm^−1^), and C–S (1320 cm^−1^). C–C–O (1320 cm^−1^), C–O–C (1160 cm^−1^), C–N (1060 cm^−1^), and CH_2_ (950, 890, 766 cm^−1^).

### 3.4. Drug Release Studies of AMP and CLX by Polymer Carrier

Drug-loaded polymers were dissolved in PBS (pH 7.4) to obtain solutions at a concentration of 1 mg/mL. Subsequently, 1 mL of each solution was placed into a dialysis membrane bag with a molecular weight cut-off (MWCO) of 3.5 kDa. The dialysis bag was then immersed in a glass vial containing 44 mL of PBS. Drug release was monitored by measuring the concentration of the drug diffused into the external PBS medium. At predetermined time intervals, 0.5 mL aliquots of the surrounding PBS were withdrawn and mixed with 0.5 mL of methanol in a cuvette. The resulting samples were analyzed using UV–Vis spectrophotometry to determine the amount of drug released, based on absorbance measurements at λ = 207 nm. The drug content in the ionic conjugates, as well as the amount of drug released, was determined using the Lambert–Beer law, based on linear calibration curves generated in a 1:1 (*v*/*v*) mixture of PBS and methanol, using the specific absorbance maxima of the ionic drugs. For dual systems, the calibration curve was established using PBS/MeOH solutions containing both drugs in a 1:1 molar ratio.

### 3.5. Characterization

^1^H NMR spectra were recorded on an Agilent NMR Magnet-400 instrument (Santa Clara, CA, USA) operating at 400 MHz. Deuterated dimethyl sulfoxide (DMSO-d_6_) was used as the solvent, and tetramethylsilane (TMS) served as the external standard. Fourier-Transform Infrared (FT-IR) spectra were obtained using a Spectrum Two 1000 instrument (PerkinElmer, Waltham, MA, USA) in attenuated total reflection (ATR) mode. The number-average molecular weight (M_n_) and dispersity index (Ð) were determined by size exclusion chromatography (SEC) using an Ultimate 3000 system equipped with a RefractoMax 521 differential refractometer (Thermo Fisher Scientific, Waltham, MA, USA). CLX-based polymer samples were dissolved in DMF containing 10 mM LiBr and analyzed at 50 °C. AMP-based and dual-drug polymer samples were dissolved in deionized water and measured at 40 °C. Separation was performed using a TSKgel Guard SuperMultiporeHZ-H pre-column (6 µm, 4.6 × 20 mm) and two TSKgel SuperMultiporeHZ-H analytical columns (6 µm, 4.6 × 150 mm) at flow rates of 0.18 mL/min (DMF) and 0.3 (water) mL/min. The system was calibrated with poly(ethylene oxide) (PEO) standards ranging from 982 to 969,000 g/mol. For MIs, the SEC was performed in THF (1100 Agilent 1260 Infinity with differential refractometer MDS RI detector, Agilent Technologies, Santa Clara, CA, USA) with an autosampler using a pre-column guard (5 µm, 7.5 × 50 mm) and two PLgel MIXED-C and MIXED-D columns (5 µm, 7.5 × 300 mm). Measurements were carried out at 35 °C, with a flow rate of 1.0 mL/min. Calibration was based on linear polystyrene standards (580–3,000,000 g/mol). Throughout the drug release process, samples extracted at specified time intervals were assessed using Ultraviolet–Visible (UV–Vis) on an Evolution 300 spectrometer (Thermo Fisher Scientific, Waltham, MA, USA). The quartz cells were used to quantify the drug content and the amount of released pharmaceutical anions. The water contact angle (WCA) of the polymeric coatings was measured using a Data Physics OCA 15EC goniometer (DataPhysics Instruments GmbH, Filderstadt, Germany) via the sessile drop method. Polymeric thin films were prepared using the spin-coating technique on 22 mm × 22 mm glass substrates. The glass plates were pre-cleaned with Hellmanex™ solution and hot water, followed by rinsing with acetone. Spin-coating was performed using a Laurell WS-650MZ spin coater (Laurell Technologies Corp., North Wales, PA, USA). A volume of 20 μL of the polymer solution was deposited onto each glass substrate and spun at a rotational speed of 1800 rpm. Goniometer measurements were carried out using SCA20 software (https://sca20.software.informer.com/). To ensure reproducibility, three independent 6 μL water droplets were deposited on each coated surface. The reported WCA values represent the average of these three measurements. Hydrodynamic diameter and polydispersity index of polymer particles were determined using dynamic light scattering (DLS). Measurements were conducted with a Nanotrac Flex analyzer equipped with a Microtrac MRB laser particle size detector (Microtrac Retsch GmbH, Haan, Germany), using Dimensions LS software (v1.1.0). An external “dip-in” probe operating at a 180° backscattering angle was used. Polymer samples were dissolved in deionized water at a concentration of 1.0 mg/mL. The results represent the mean values obtained from three independent measurements.

## 4. Conclusions

Graft copolymers, synthesized via controlled radical polymerization using pharmaceutically active TMAMA-based IL monomers, were yielded as well-defined structures with IL contents ranging from 20 to 60 mol% in the side chains. These DDS, containing one or two types of pharmaceutical anions, that is cloxacillin (CLX) and/or ampicillin (AMP), were designed to evaluate the drug content and release behavior. The side-chain length (DP_sc_ = 21–60) was controlled by monomer conversion, while the ionic content (F_TMAMA_ = 12–55 mol%) was adjusted through feed composition. Copolymers conjugated with CLX anions exhibited a lower ionic content than the initial TMAMA feed ratio, whereas AMP conjugates and dual-drug systems retained F_TMAMA_ values similar to the feed composition. Similarly, in dual systems, the CLX content was lower than AMP (F_TMAMA/CLX_/F_TMAMA_ ≈ 30–38%), indicating the lower reactivity of TMAMA/CLX than TMAMA/AMP.

The resulting nanocarriers exhibited hydrophilic surfaces (WCA = 30–54°) and particle diameters ranging from 90 to 360 nm. Ionically bound drug content ranged from 27 to 65% for CLX and 48–70% for AMP in single-drug systems and 47–79% in dual-drug systems. In vitro release followed a biphasic pattern: an initial burst phase (within 1 h, releasing 23–79% of the drug), followed by sustained release over 72 h. Final drug concentrations reached 5–16 µg/mL (CLX), 12–13 µg/mL (AMP), and 10–15 µg/mL (CLX + AMP). CLX exhibited a lower affinity for the polymer rich in ionic content and was released more rapidly during the burst phase compared to AMP. The drug release from dual-drug systems was more hindered, likely due to stronger ionic interactions between the drugs themselves and between the drugs and the polymer compared to the single-drug systems.

Overall, the study demonstrates that cholinium-based IL monomers enable the development of advanced single- and dual-drug DDS with tunable architectures and the effective potential for antibiotic co-delivery.

## Figures and Tables

**Table 1:** Characteristics of graft copolymers carrying anions of AMP or CLX.

No.	[TMAMA: MMA]_0_	DG [%]	^1^H NMR			SEC		UV-Vis
			DP_sc_	F_TMAMA_ [mol%]	M_n,NMR_ ·10^−3^ [g/mol]	M_n,SEC_ ·10^−3^ [g/mol]	Ð	DC [%]
A1	25:75	28	44	24	284.1	98.5	1.60	52
C1			56	11	277.0	512.7	1.25	27
A3	50:50		57	44	483.1	166.6	1.63	62
C3			21	30	172.8	583.3	1.22	65
A2	25:75	48	30	22	316.0	90.0	1.56	48
C2			60	14	600.0	530.1	1.28	31
A4	50:50		52	52	820.2	410.0	1.52	70
C4			29	23	322.0	546.5	1.19	62

Where M:MI = [TMAMA+MMA]_0_:[MI]_0_ = 200:1, [MI]_0_:[CuCl]_0_:[bpy]_0_ = 1:1:2, MEOH:TMAMA (v:wt) = 4:1 (series A) and 3:1 (series C), and MeOH:THF (v:v) = 4:3 (series A) and 3:2.25 (series C), reaction time of 20 h. DP_sc_—polymerization degree of the side chains, F_TMAMA_—content of TMAMA in the side chains, and SEC—determined in water for AMP conjugates or DMF for CLX conjugates, PEO calibration.

**Table 2:** Characteristics of graft copolymers carrying anions of AMP and CLX.

No.	[TMAMA/AMP: TMAMA/CLX: MMA]_0_	DG [%]			^1^H NMR		SEC		UV-Vis
			DP_sc_	DP_TMAMA/AMP_/DP_TMAMA/CLX_	F_TMAMA/CLX_/F_TMAMA/AMP_ [mol%]	M_n,NMR _ ·10^−3^ [g/mol]	M_n,SEC _ ·10^−3^ [g/mol]	Ð	DC [%]
AC1	12.5:12.5:75	28	27	4/2	7/15	180.3	437.8	1.76	47
AC3	25:25:50		44	13/7	14/32	395.2	473.1	1.75	74
AC2	12.5:12.5:75	48	39	5/3	8/13	397.0	636.8	1.76	61
AC4	25:25:50		37	13/7	19/36	627.6	456.0	1.43	79

Where M:MI = [TMAMA+MMA]_0_:[MI]_0_ = 200:1, [MI]_0_:[CuCl]_0_:[bpy]_0_ = 1:1:2, MEOH:TMAMA (v:wt) = 3.75:1 (AC1–AC2) and 4:1 (AC3–AC4), and MeOH:THF (v:v) = 3.75:2.8 (AC1–AC2) and 4:3 (AC3–AC4), reaction time of 20 h. DP_sc_—polymerization degree of the side chains, F_TMAMA_—content of TMAMA in the side chains, and SEC—determined in water, PEO calibration.

## Data Availability

Data is contained within the article and Supplementary Materials.

## Supplementary Materials

The following supporting information can be downloaded at: https://www.mdpi.com/article/10.3390/ijms26199415/s1.
