# Supplementary material for: Graft Polymers Derived from Pharmaceutically Active Choline-Based Ionic Liquid Monomers: Dual Incorporation of Ampicillin and Cloxacillin
Source: Int J Mol Sci. 2025 Sep 26;26(19):9415. doi: 10.3390/ijms26199415 (PMC12524462; doi:10.3390/ijms26199415)

Supplementary Materials

Graft Polymers Derived from Pharmaceutically Active Choline-Based Ionic Liquid Monomers: Dual Incorporation of Ampicillin and Cloxacillin

Aleksy Mazur [1], Dorota Neugebauer [1]

[1]Department of Physical Chemistry and Technology of Polymers, Faculty of Chemistry, Silesian University of Technology, 44-100 Gliwice, Poland; Aleksy.Mazur@polsl.pl (A.M.)

* Correspondence: Dorota.Neugebauer@polsl.pl (D.N.)

Content:

Procedure S1. Synthesis of MI.

Table S1. Particle sizes determined by DLS analysis.

Table S2. Hydrophilicity of polymeric layers.

Table S3. Data for drugs released from polymeric carriers.

Table S4. Parameters of kinetic model equations.

Figure S1. FT-IR spectrum of single drug conjugates.

Figure S2. Kinetics profiles by various models for release of a) AMP, b) CLX, c) AMP and CLX.

Figure S3. SEC chromatograms of polymers.

Figure S4. ^{1}H NMR spectra of AMP based ionic conjugates in reaction mixtures (series A). Signal symbols are corresponding to those in the Figure 2a, Integration for the pyrene signal set to 1.a

Figure S5. ^{1}H NMR spectra of CLX based ionic conjugates in reaction mixtures (series C). Signal symbols are corresponding to those in the Figure 2a, Integration for the pyrene signal set to 1.

Figure S6. ^{1}H NMR spectra of AMP/CLX based ionic conjugates in reaction mixtures (series AC). Signal symbols are corresponding to those in the Figure 2a, Integration for the pyrene signal set to 1.

Procedure S1. Synthesis of MI.

Synthesis of MI-1: The macronitiator with smaller content of bromoester groups was obtained using the following initial proportions $[HEMA]_0$:$[MMA]_0$:$[EBiB]_0$:$[CuBr]_0$:$[dNbpy]_0$ = 150:450:1:1:2. According to the standard procedure, comonomers HEMA (2 mL, 16.4 mmol) and MMA (5.2 mL, 49.3 mmol), anisol (0.7 mL), dNbpy (47.27 mg, 0.11 mmol) and CuBr catalyst (15.7 mg, 0.11 mmol) were placed into a Schlenk flask and degassed by two freeze–pump–thaw cycles. The initial sample was taken and EBiB initiator (16.3 µl, 0.11 mmol) was introduced to the mixture. Next, the reaction flask was immersed in an oil bath at 70 ºC. The reaction was stopped after 2 hours by exposing to air. The polymer solution in THF was passed through a neutral alumina column to remove copper catalyst, then precipitated in diethyl ether and vacuum dried. Obtained polymer (700 mg, including 1.56 mmol of HEMA units) was dissolved in pyridine (8 mL). Next, the mixture was placed in an ice bath to cool it down to 0 ºC. After cooling the esterification agent BiBB (580 µL, 4.6 mmol) was added dropwise. The mixture was stirred overnight at 20 ºC. Next, the bromoester-functionalized polymer was precipitated in cooled water and vacuum dried.

Synthesis of MI-2: The macronitiator with larger content of bromoester groups was obtained according to the above procedure using the following initial proportions $[HEMA]_0$:$[MMA]_0$:$[EBiB]_0$:$[CuBr]_0$: $[dNbpy]_0$ = 300:300:1:1:2. Polymerization: comonomers HEMA (2 mL, 16.4 mmol) and MMA (1.75 mL, 16.4 mmol), anisol (0.4 mL), dNbpy (44.80 mg, 0.11 mmol), CuBr catalyst (7.9 mg, 0.055 mmol), EBiB initiator (8.2 µl, 0.055 mmol). The reaction was stopped after 0.5 hour by exposing to air. Esterification: the obtained polymer (270 mg, including 1.04 mmol of HEMA units), pyridine (6 mL), BiBB (385 µL, 3.1 mmol).

Table S1. Particle sizes determined by DLS analysis.

No.	Intensity [%]	D_H [nm]	PDI
A1	100	194	0.01
A2	100	192	0.006
A3	100	226	0.005
A4	90	225	0.01
	10	1700	
C1	14	57	0.3
	86	270	
C2	96	178	0.007
	4	1186	
C3	100	359	0.2
C4	88	318	0.04
	12	1298	
AC1	100	201	0.01
AC2	40	94	0.17
	60	183	
AC3	33	130	0.27
	66	300	
AC4	26	92	0.25
	74	300	

where: D_H is hydrodynamic diameter and PDI is polydispersity index.

Table S2. Hydrophilicity of polymeric layers.

No.	WCA [°]
A1	54.0±1.2
A2	50.0±1.5
A3	39.0±0.6
A4	30.0±0.6
C1	45.0±3.0
C2	45.0±3.1
C3	41.0±0.6
C4	35.0±1.7
AC1	50.0±2.5
AC2	53.0±1.0
AC3	40.0±2.1
AC4	40.0±1.2

where: WCA is water contact angle.

Table S3. Data for drugs released from polymeric conjugates.

No.	CRD [µg/mL]	ARD [%]
A1	12.6±0.8	92±6
A2	12.4±0.8	98±6
A3	12.2±0.8	74±5
A4	12.8±0.8	69±5
C1	5.0±0.3	70±4
C2	6.5±0.3	80±3
C3	15.9±0.3	93±2
C4	14.3±0.3	85±2
AC1	8.8±0.4	71±4
AC2	9.8±0.5	61±2
AC3	13.2±0.4	68±3
AC4	15.2±0.5	73±3

where: CRD is concentration of released drug and ARD is percentage amount of released drug.

Table S4. Parameters of kinetic model equations.

No.	Zero order<br>$ARD = K_0 \times t$	First order<br>$\ln(1\text{-}ARD) = K \times t$	Higuchi<br>$ARD = K_H \times t^{0.5}$	Korsmeyer-Peppas<br>$ARD/ARD_t = K_{K\text{-}P} \times t^n$	
	R^2	R^2	R^2	R^2	n
A1	0.64	0.84	0.79	0.94	0.30
A2	0.32	0.77	0.45	0.91	0.27
A3	0.86	0.92	0.94	0.96	0.25
A4	0.67	0.80	0.82	0.96	0.57
C1	0.47	0.62	0.60	0.91	0.48
C2	0.46	0.64	0.60	0.93	0.47
C3	0.62	0.81	0.78	0.97	0.23
C4	0.55	0.72	0.70	0.91	0.29
AC1	0.35	0.40	0.49	0.90	0.19
AC2	0.58	0.65	0.70	0.92	0.21
AC3	0.68	0.79	0.82	0.98	0.39
AC4	0.75	0.85	0.89	0.94	0.31

where: ARD is amount of drug released at time t, ARD_t is total amount of drug released, K is the release rate constant, R^2 is the correlation coefficient, n is the diffusion exponent.

Figure S1. FT-IR spectrum of single drug conjugates.

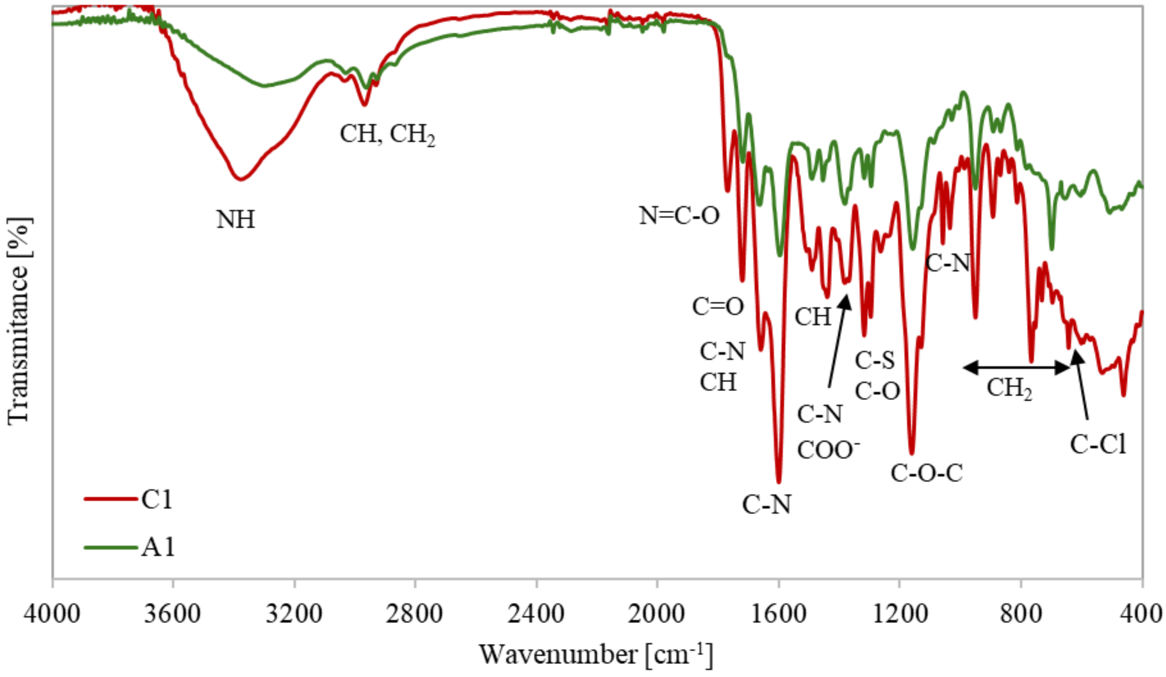

Figure S2. Kinetics profiles by various models for release of a) AMP, b) CLX, c) AMP and CLX.

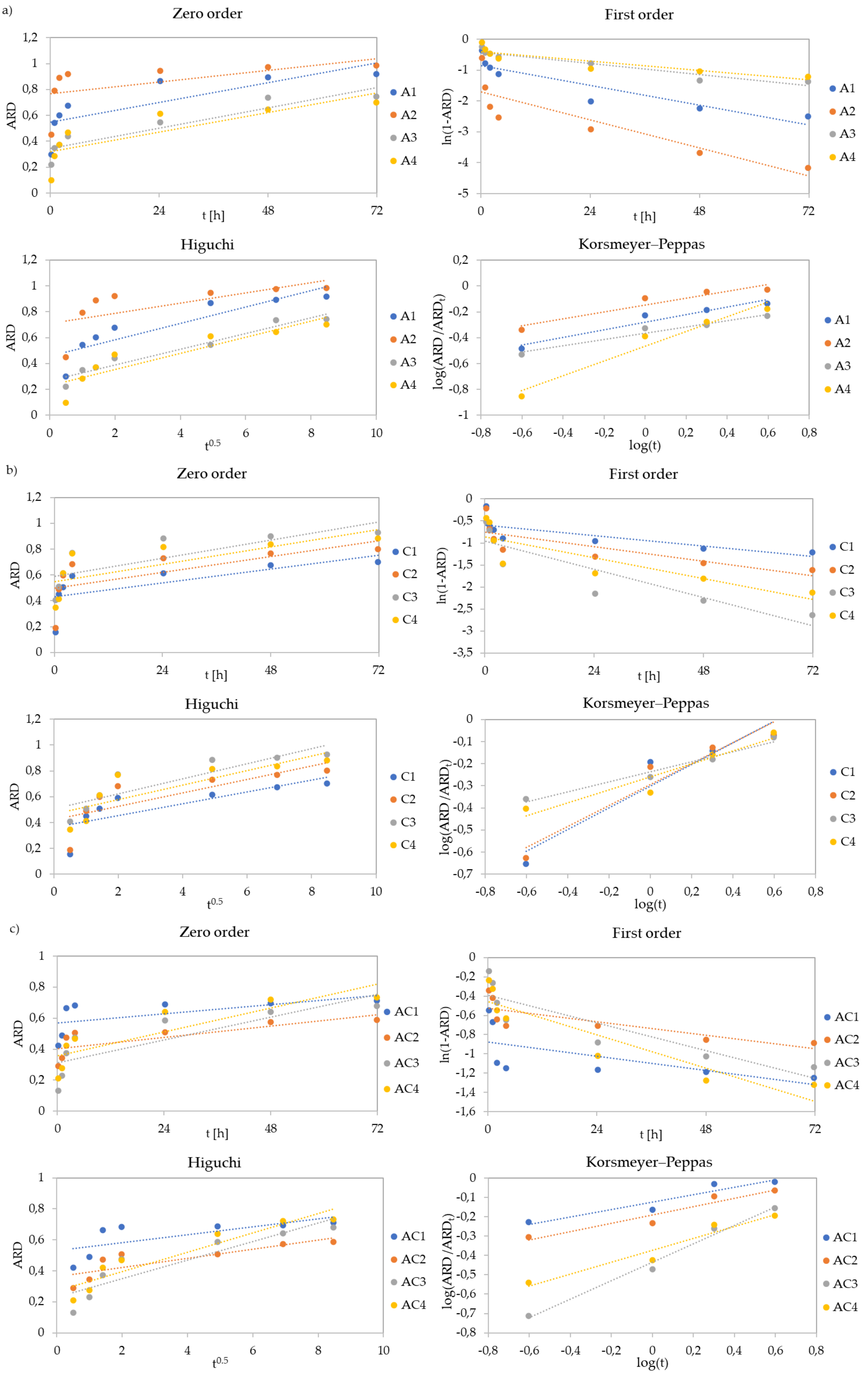

Figure S3. SEC chromatograms of polymers (series A, C, AC, and MIs).

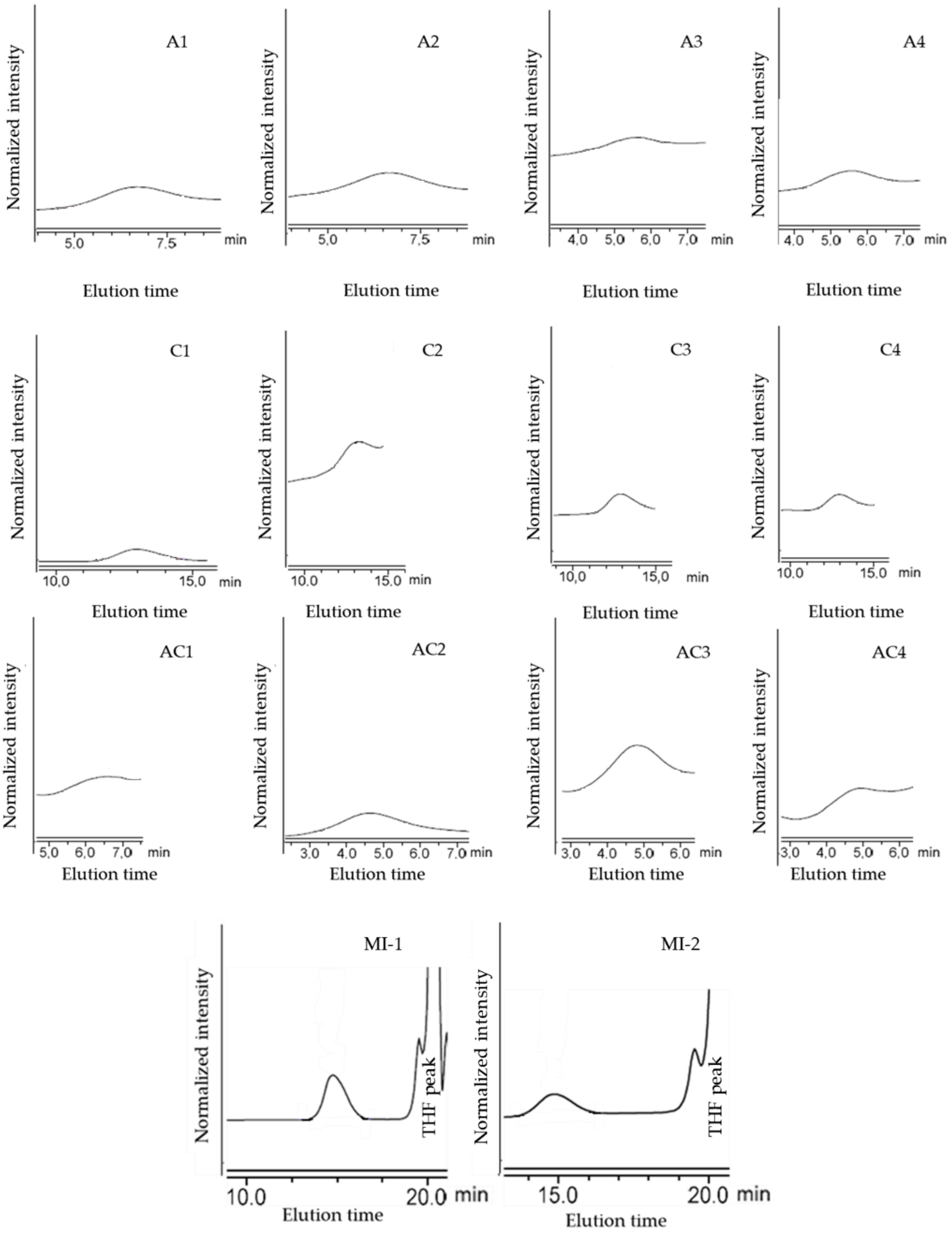

Figure S4. ^{1}H NMR spectra of AMP based ionic conjugates in reaction mixtures (series A). Signal symbols are corresponding to those in the Figure 2a, Integration for the pyrene signal set to 1.

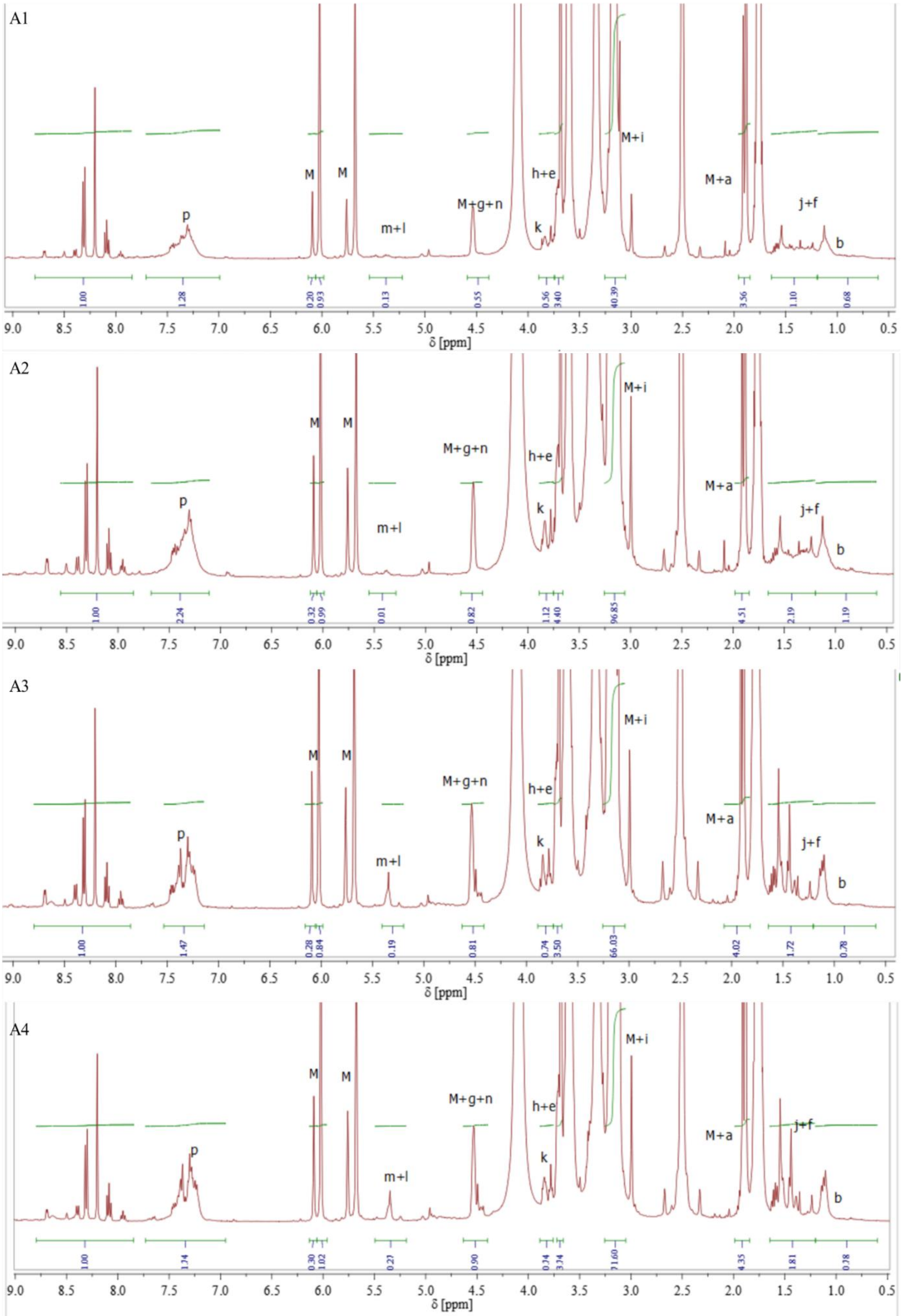

Figure S5. ^{1}H NMR spectra of CLX based ionic conjugates in reaction mixtures (series C). Signal symbols are corresponding to those in the Figure 2a, Integration for the pyrene signal set to 1.

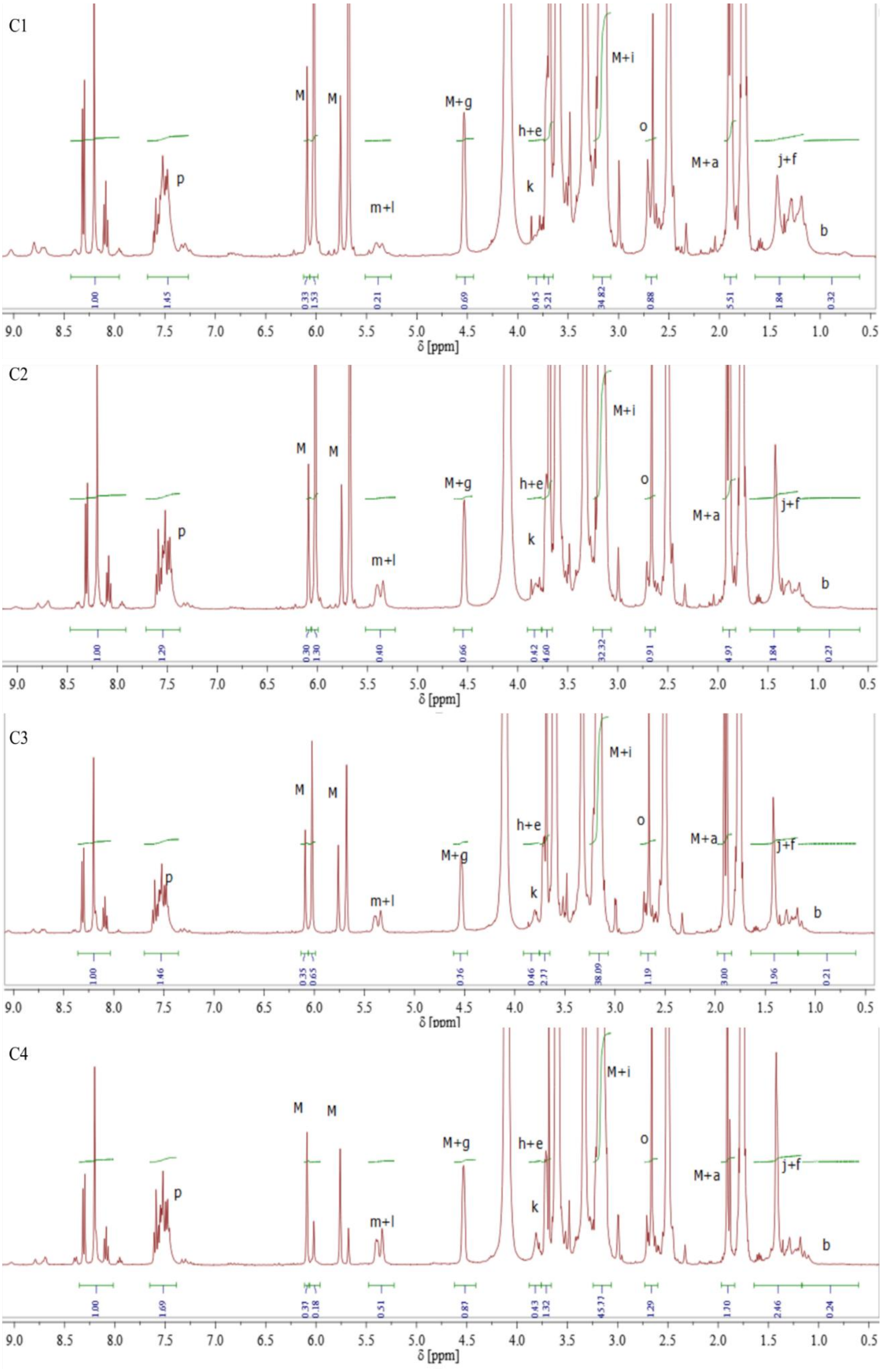

Figure S6. ^{1}H NMR spectra of AMP/CLX based ionic conjugates in reaction mixtures (series AC). Signal symbols are corresponding to those in the Figure 2a, Integration for the pyrene signal set to 1.

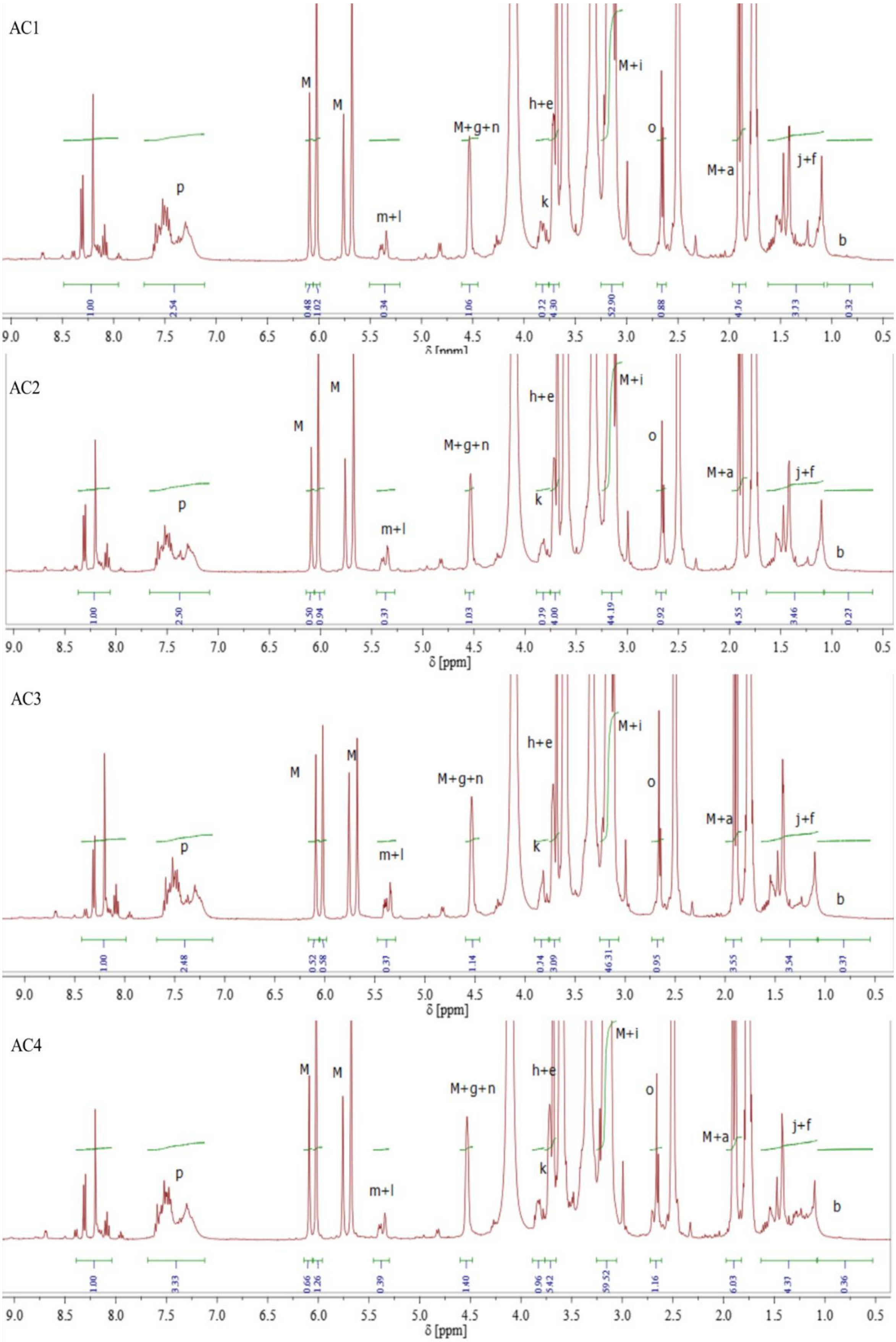

Supplement: Supplementary file 1 [file ijms-26-09415-s001.zip › ijms-3803885-supplementary.pdf]
